# Spontaneous Retroperitoneal Hematoma After Total Hip Arthroplasty

**DOI:** 10.7759/cureus.38971

**Published:** 2023-05-13

**Authors:** Alexander M DeLeon, Mukund Gande, Vicente Garcia Tomas

**Affiliations:** 1 Anesthesiology, Northwestern University Feinberg School of Medicine, Chicago, USA

**Keywords:** femoral nerve, neurologic deficit, total hip arthroplasty, spinal anesthesia, retroperitoneal hematoma

## Abstract

Spontaneous retroperitoneal hematomas are a rare yet potentially devastating occurrence associated with antiplatelet and anticoagulant therapies. We present a case of a spontaneous retroperitoneal hematoma post-operatively after a total hip arthroplasty surgery performed under a midline approach spinal anesthetic. A 79-year-old male with a BMI of 25.72 kg/m^2^ presented for anterior total hip arthroplasty. A midline approach with an uncomplicated spinal anesthetic was performed. On the night of postoperative day 0, the patient received a prophylactic dose of dalteparin. The patient reported back pain, contralateral leg numbness, and weakness that began overnight on postoperative day 0. A computed tomography (CT) scan confirmed a 10 cm, contralateral retroperitoneal hematoma. The patient underwent interventional radiology embolization followed by surgical evacuation and demonstrated improvement in the neurologic function of his affected leg. Despite the rarity of a spontaneous retroperitoneal hematoma formation in the perioperative period, it could be simultaneously evaluated when performing an MRI to rule out spinal hematoma if a patient suffers a post-op neurologic deficit after a neuraxial technique. Understanding the evaluation and timely treatment of patients at risk for a perioperative retroperitoneal hematoma could help clinicians prevent a permanent neurologic deficit.

## Introduction

Spontaneous retroperitoneal hematomas (SRH) have been reported in the literature and are linked to antiplatelet and anticoagulant therapy [1]. Retroperitoneal hematomas (RH) are a known complication of specific regional anesthetic procedures, including lumbar plexus blocks with and without associated systemic anticoagulation therapy [2-5]. The presentation of an SRH is nonspecific, and diagnosis could be delayed following a neuraxial procedure if not considered in the differential diagnosis for an acute perioperative neurologic deficit [1]. We report a case of a contralateral symptomatic SRH following a total hip arthroplasty surgery performed under neuraxial anesthesia, presenting with back pain and a neurologic deficit. Knowledge of the potential for this occurrence could aid in the timely diagnosis and management before permanent complications result. The CARE checklist and guidelines were used to prepare this manuscript.

## Case presentation

A 79-year-old, 5'7", 74.5 kg (BMI 25.72 kg/m^2^) male with a past medical history significant for non-obstructive coronary artery disease, mild asthma, hypertension, and chronic renal insufficiency was scheduled for a left-sided anterior total hip arthroplasty with spinal anesthesia. His medications included valsartan and fenofibrate. The spinal anesthetic was performed at 12:25 p.m. with a 25-gauge 8.9 cm PENCAN spinal needle (B. Braun, Bethlehem, PA) via midline approach at the L3-4 level with a single attempt requiring no needle redirections. The local anesthetic injected was 13 mg of 0.5% isobaric bupivacaine. Surgery concluded at 2:40 p.m. At midnight, the night of surgery, the patient received 2,500 units of dalteparin subcutaneously (SQ) for venous thromboembolism (VTE) prophylaxis. Overnight on postoperative day (POD) 0, the patient began complaining of right hip pain radiating from the back of the patient to the non-operative right knee. The Hb was 7.8 g/dL, compared to 16.2 g/dL two months before surgery. The patient's vital signs were stable, with no evidence of acute hypovolemia.

**Figure 1:**
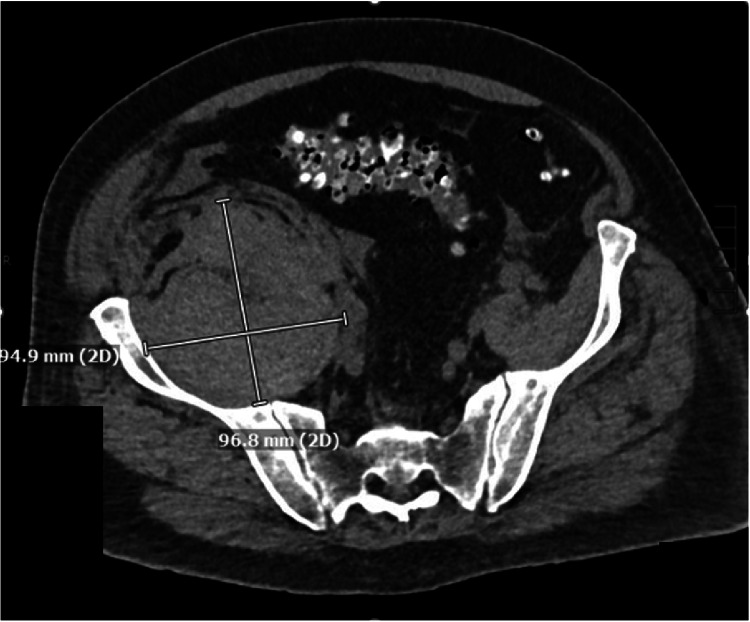
Computed tomography (CT) scan. A right-sided 96.8 mm by 94.9 mm spontaneous retroperitoneal hematoma is visible.

**Figure 2:**
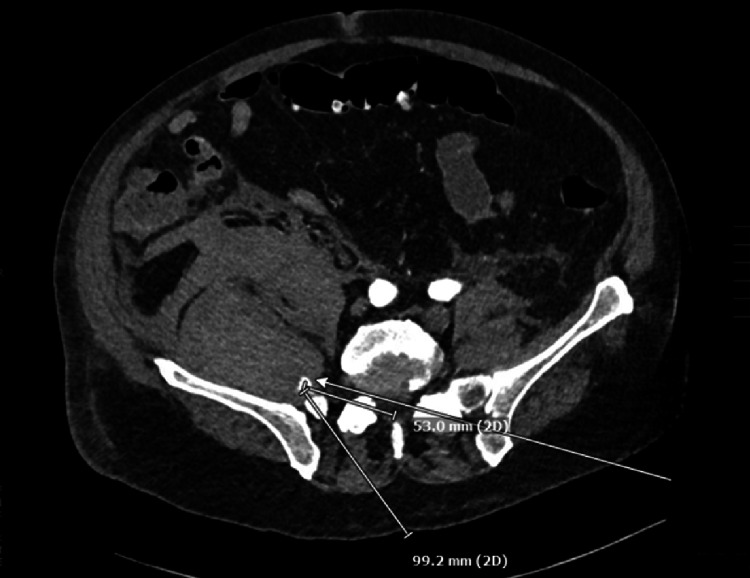
Computed tomography (CT) scan. The "contrast blush" sign (arrow) demonstrates the location of the source of the bleed.

The neurologic exam demonstrated right leg numbness and weakness in the hip and knee flexors in the femoral nerve distribution. On POD one, a lumbar spine MRI was obtained to rule out a complication related to the spinal anesthetic. The exam showed no epidural hematoma; however, a large right-sided RH was partially visualized. On POD two, a computed tomography (CT) angiogram of the abdomen and pelvis was performed to visualize the RH better and assess for progression. A 96.8 mm by 94.9 mm RH was characterized as being centered within the right iliopsoas musculature (see Figure 1). The CT scan also revealed a partially calcified right common iliac artery with an associated fusiform aneurysmal dilatation measuring 1.8 cm. A "contrast blush" within the right iliacus, possibly arising from the proximal right internal iliac artery (see Figure 2).

On POD two, the patient underwent successful coil embolization of a pseudoaneurysm arising from a proximal iliolumbar branch from the internal iliac artery. Due to persistent symptoms, on POD three, the patient underwent hematoma evacuation and drainage, and approximately 400 mL of hematoma was evacuated. The patient was discharged on POD nine with improving motor and sensory function in the right femoral nerve distribution to receive inpatient rehabilitation.

At a four-month follow-up, after intensive physical therapy, the patient noted significant improvement in the strength and sensation of his right leg.

## Discussion

An RH is spontaneous if it is not caused by an invasive procedure, trauma, surgery, or abdominal aortic aneurysm [1]. Retroperitoneal hematomas may manifest as hemodynamic instability, new-onset neurologic deficits, or pain not explained otherwise [6-8]. Procedures such as lumbar plexus blockade have been reported to be associated with RHs [2,3,5,9]. No lumbar plexus block was performed in our patient. The use of anticoagulation has also been associated with the development of RHs [2,5,10]. Our patient received perioperative anticoagulation per routine for VTE prophylaxis for all total hip arthroplasty patients.

Two cases of RHs after lumbar punctures (LPs) have been reported in the literature [11,12]. In one case, the patient underwent an LP with a 22-gauge needle in the left-lateral decubitus position. Multiple attempts and multiple providers were involved. The patient became hemodynamically unstable with a BP of 70/40 and a hematocrit decline from 41% to 20%. The diagnosis was made by a CT scan showing a "massive" hematoma from T12 to L5. The second case involved an LP performed in the left-lateral decubitus position using a 22-gauge needle. The patient had received 5000 units of SQ heparin 18 hours before the procedure. Multiple lumbar puncture attempts were required. The patient went into hypovolemic shock, requiring resuscitation and interventional radiology embolization.

A single case of an RH occurring after spinal anesthesia has been reported [13]. In that case, the patient had an elevated BMI of 33.3 kg/m^2^, and a 22-gauge Quincke needle was inserted via the paramedian approach for right toe amputation. The needle was reportedly inserted almost to its full length during a failed attempt without cerebrospinal fluid detection. The patient reported back pain and quadriceps weakness while still in the post-anesthesia care unit (PACU) after the 65-minute amputation procedure. A CT scan revealed a large RH. The bleeding vessel was identified as a lumbar artery at the level of L2 and L3 through the "contrast blush" sign (indicating the bleeding vessel), coinciding with the location of the spinal anesthetic attempts.

In contrast to the previously reported cases, the spinal anesthetic did not cause the RH in the current case report. A single attempt at midline placement, with successful CSF confirmation and surgical anesthesia, confirmed the spinal needle's location.

RHs have been associated with total hip arthroplasty surgery [7,14]. Both cases of RH related to total hip arthroplasty surgery occurred on the ipsilateral side, and our reported case happened on the contralateral side. No surgical manipulation of the contralateral limb occurred intra- or post-operatively.

Our patient did have vascular abnormalities, such as a "partially calcified dissection of the proximal/mid right common iliac artery with associated fusiform aneurysmal dilatation measuring 1.8 cm." The vascular abnormality was noted during the workup for the retroperitoneal hematoma, yet it was likely a chronic finding given the partial calcification. Thus, the pre-existing vascular disease might have contributed to the SRH.

Risk factors for SRHs include age >70, the intensity of anticoagulation, and antiplatelet agents [15]. Our patient was >70 years old. He received a dose of prophylactic dalteparin the night he developed RH symptoms, per the usual routine. Yet, he received no other medication, such as coumadin or aspirin, which would have made a spontaneous bleed more likely [7,14].

Our patient presented with neurologic symptoms, including pain, numbness, and weakness in the distribution of the lumbar plexus. Initial concerns were for a central lesion such as a spinal hematoma, and a timely MRI study ruled out a spinal hematoma. The unexpected finding of the SRH on POD one hastened the eventual definitive diagnosis and surgical treatment. The decision to evacuate the hematoma was made due to the patient's neurologic status not improving after the lesion was embolized by interventional radiology. The patient's right leg pain and neurological exam improved after surgical treatment.

## Conclusions

SRH should be considered when compatible new-onset neurologic symptoms are present in the perioperative period in the presence of risk factors. Risk factors for SRH include age >70, anticoagulation use, and the use of antiplatelet agents. The rarity of SRH, yet the potentially devastating nature both hemodynamically and neurologically, should lead clinicians practicing perioperative medicine to understand the presentation, risk factors, diagnosis, and treatment.
